# Contradictory Symptoms

**Published:** 1888-06

**Authors:** 


CONTRADICTORY SYMPTOMS.—Every remedy has some symptoms contradictory to the general action of the drug; thus the Arsenic headache is better from cold and the Pulsatilla colic, from heat.

